# Vaccination With Live Attenuated Vaccines in Four Children With Chronic Myeloid Leukemia While on Imatinib Treatment

**DOI:** 10.3389/fimmu.2020.00628

**Published:** 2020-04-17

**Authors:** Claudia Bettoni da Cunha-Riehm, Verena Hildebrand, Michaela Nathrath, Markus Metzler, Meinolf Suttorp

**Affiliations:** ^1^Department of Pediatric Hematology and Oncology, Medical School Hannover, Hanover, Germany; ^2^Department of Pediatric Hematology and Oncology, University Hospital Erlangen, Erlangen, Germany; ^3^Department of Pediatric Hematology and Oncology, Klinikum Kassel, Kassel, Germany; ^4^Medical Faculty, Pediatric Hemato-Oncology, Technical University Dresden, Dresden, Germany

**Keywords:** imatinib, tyrosine kinase inhibitors, mild immunosuppression, chronic myeloid leukemia, pediatric CML, vaccination, measles, varicella

## Abstract

Chronic myeloid leukemia (CML) in childhood and adolescence is a rare malignancy that can successfully be treated with the tyrosine kinase inhibitor (TKI) imatinib. According to the current experience, treatment is necessary for years and, in the majority of cases, a lifelong approach is required to control the malignant disease. To what extent imatinib causes immunosuppression in different age cohorts is a controversial discussion. According to general medical recommendations, live vaccines are contraindicated in individuals treated with imatinib. However, a recent increase in the number of globally reported cases of measles has been observed and continues to rise. Due to the high contagiousness of the virus, near-perfect vaccination coverage (herd immunity of 93 to 95%) is required to effectively protect against measles resurgence—a scenario that is not realistic in many countries. When four teenagers with CML (median age 13 years, range 12–15) who were enrolled into pediatric trial CML-paed II while on imatinib treatment (median treatment duration 36 months, range 11–84) were identified without protective measles and/or varicella titers, we carefully balanced the risks of a live vaccination under immunosuppressive TKI medication against the benefit of being protected. The patients underwent live vaccination with the live attenuated vaccines M-M-RVAX Pro^®^ and Varivax^®^ simultaneously (Patient #1), Priorix^®^ and Varilix^®^ consecutively (Patient #2), and Priorix^®^ (Patients #3 and #4). While the first three patients were vaccinated while receiving TKI therapy, treatment with imatinib was interrupted in patient #4 for 1 week prior and 2 weeks after vaccination. Patients #1 and #3 reacted with stable long-term seroconversion. In Patient #2, serum titer conversion against measles and varicella could not be demonstrated and thus revaccination with Priorix^®^ and Varilix^®^ was performed 3 years later. However, protective titers did not develop or were lost again. Patient #4 also lost protective titers against measles when assessed 10 months after vaccination, but revaccination resulted in stable seroprotective titers over 12 months after the last vaccination during ongoing imatinib treatment. We conclude that in all patients, the safety of live vaccines could be documented, as no acute or late adverse events were observed. However, in line with observations that memory B-cells are lost under exposure to imatinib, revaccination may become necessary (two out of four patients in this small series lost their seroprotection). Considering that the number of cases is very small, we also suggest some criteria for decision-making regarding live vaccinations of CML patients treated with imatinib.

## Introduction

Chronic myeloid leukemia (CML) in childhood and adolescence is a rare malignancy, representing only 2–3% of pediatric leukemias, with an annual incidence of 1 case per million in the first two decades of life in Western countries (1). The Philadelphia chromosome—a reciprocal balanced translocation *t*(9;22)(q34;q11)—is the genetic hallmark of the disease. The resulting gene fusion encodes for a chimeric BCR-ABL1 fusion protein representing a constitutively active tyrosine kinase (2). The development of the drug imatinib two decades ago, acting as a targeted tyrosine kinase inhibitor (TKI) at the ATP-binding pocket of BCR-ABL1, has revolutionized the treatment of CML (3). By oral medication, all clinical signs of CML can be resolved, and allogeneic stem cell transplantation (alloSCT), previously the single curative option, can now be reserved for particular conditions for almost all adults as well as pediatric patients diagnosed with the chronic phase (CML-CP). Once shown effective, imatinib and successive next-generation TKIs have converted CML-CP from a life-threatening blood cancer to a manageable chronic disease (4–7). Adults with CML-CP now can expect a lifespan identical to that of the general population (8). Also, discontinuing TKIs in adult patients after having achieved a deep and sustained molecular response (MR) after several years of treatment has proven feasible (9, 10).

The situation for minors is more complex (11). Because children have not finished growing, they face unique side effects not seen in adults during TKI administration (longitudinal growth retardation, delayed dentition, problems with treatment adherence during adolescence) (5, 12–14). Concerns of a life-long treatment render cessation of treatment likely to be more beneficial in terms of reducing TKI-related side effects (15). However, in pediatrics only, limited data so far are available, and the rarity of the disease makes evidence-based recommendations difficult (4, 5).

Different first- (imatinib), second- (nilotinib, dasatinib, and bosutinib), and third-generation TKIs (ponatinib) are used to treat CML without precise knowledge as to what extent these drugs affect the immune system. Data from *in vitro* and animal studies have documented seemingly contradictory effects of imatinib. In adults on imatinib treatment compared to adult patients with CML on other treatments, a low frequency of varicella-zoster virus (VZV) infections (15 episodes of herpes zoster and 1 varicella, in 771 patients) has been described (16). The authors concluded that VZV infection is more frequent with longer duration of CML disease and with prior therapy. VZV infection, in general, can be cured with acyclovir antiviral therapy, does not disseminate, and does not mandate a recommendation for antiviral drug administration at a prophylactic dosage in such patients. Data on VZV infection in children with CML are not available. *In vitro*, imatinib inhibited T-cell receptor-mediated activation of human T cells dose-dependently (17), while *in vivo* imatinib treatment in mice selectively inhibited the expansion of antigen-experienced memory cytotoxic T-cells (CTL) without affecting primary T- or B-cell responses (18). Therefore, the authors concluded that imatinib may be efficacious in the suppression of CTL-mediated immunopathology in autoimmune diseases, without the risk of acquiring viral infections.

On the other hand, in a 54-year-old man with chronic hepatitis B virus (HBV) infection and CML treated with imatinib, a fatal course of reactivation of HBV has been observed (19, 20). Thus, chronic HBV infection is listed as a warning in the package information leaflets. In a prospective trial, de Lavallade et al. investigated the *in vivo* B-cell response to vaccination against influenza and pneumococcal vaccines in 51 chronic-phase adult CML patients on imatinib, dasatinib, or nilotinib, compared to 24 controls (21). It was found that TKIs, through off-target inhibition of kinases important in B-cell signaling, reduce memory B-cell frequencies and induce significant impairment of B-cell responses. *In vitro* and *in vivo*, dasatinib also inhibited antigen-specific proliferation of murine CD4+ and CD8+ transgenic T cells (22). In line with this, immunosuppression and atypical infections were observed, in addition to other severe or life-threatening side effects in 12 out of 16 CML patients treated with a high dose of 140 mg dasatinib daily (23). It also has been described that tyrosine kinases ABL1 and the SRC family are involved in the release of virions from an infected cell (24, 25). Thus, inhibition of virus release by TKIs might even represent a novel approach to antiviral treatment. Of note, in children with CML-CP, no opportunistic infections or uncommon courses of an infection were observed in larger pediatric CML trials while patients were on imatinib treatment for several years (6, 7, 26).

According to current recommendations, all vaccination schedules with live vaccines should be completed by the age of 4 to 6 years in most countries. As CML is rarely seen in children below that age, only very few children face the issue of live vaccine administration during TKI treatment. Importantly, there are older children who have missed vaccinations and also an increasing number of parents who are refusing immunizations for their children.

Carefully balancing the risks and benefits of the general recommendations on the non-usage of live vaccines while on imatinib treatment against the advantages of being protected against vaccination-preventable diseases, we report on four teenagers with CML treated with imatinib who underwent live vaccination. Through close monitoring, the safety of live vaccines could be documented in all patients. In addition, we give an overview of the criteria that should be used to form a basis for an individual decision.

## Patients and Methods

Children younger than 18 years old who had been previously diagnosed with untreated Ph+ CML were enrolled in the study of CML-paed II. The protocol was approved by the Ethical Board of the Medical Faculty of the Technical University of Dresden (Ethical vote # EK282122006), registered with ClinicalTrials.gov (NCT00445822), and conducted in accordance with the Declaration of Helsinki. Details on the treatment applied and the side effects of TKI treatment observed were collected from participating centers using standardized forms that were filled in at diagnosis and subsequently at 3-month follow-up intervals (7).

Vaccination status was not investigated on a routine base at diagnosis of CML, and investigations on vaccination were not part of the treatment protocol CML-paed II. But during the ongoing monitoring of response to CML treatment, when the problem of seronegativity was identified by checking the vaccination status during a measles outbreak in an individual patient’s surrounding area, attempts were undertaken to discuss and balance, on an individual basis, the risk of viral infection with measles, mumps, rubella, or varicella virus against the risks of vaccination against these diseases with a live virus vaccine. The legal caregivers (parents in all cases) and the patients who received live vaccination gave written informed consent after detailed counseling on all aspects of this approach. Vaccines used were identical with vaccines used on a routine basis in vaccination procedures and comprised Hexavac^®^ (Aventis Pasteur MSD, inactivated diphtheria-, tetanus-, pertussis-, poliomyelitis-, hepatitis B-, and hemophilus influenzae type b-vaccine), M-M-RVAX Pro^®^ (Merck Sharp Dome, live attenuated viral strains measles: Enders’ Edmonston; mumps: Jeryl-Lynn; rubella: Wistar RA 2773), Priorix^®^ (Glaxo Smith Kline, live attenuated viral strain measles: Schwarz; mumps: RIT 4385 derived from Jeryl Lynn strain; rubella: Wistar RA 27/3), Priorix-Tetra^®^ (Glaxo Smith Kline, live attenuated viral strain measles: Schwarz; mumps: RIT 4385 derived from Jeryl Lynn strain; rubella: Wistar RA 27/3, varicella: OKA), Varilix^®^ (Glaxo Smith Kline, live attenuated varicella strain: OKA), and Varivax^®^ (Merck Sharp Dome, live attenuated varicella strain: Oka/Merck). Details on the brand of vaccine administered in the four individual cases are shown in the “Results” section.

All patients were vaccinated intramuscularly as outpatients and thereafter dismissed under the stipulation of seeking medical advice by telephone in case of fever, local pain, or any other uncommon event and also—if suggested by the physician—to follow up physically at a medical practice. Of note, there was no specific protocol for follow-up, but it was orally recommended to asses seroconversion 3 months after vaccination. Subtypes of lymphocytes were not assessed, neither prior nor after vaccination.

### Determination of Serum Anti-viral IgG Antibodies

Titers against VZV, measles, and mumps were determined from blood serum specimen by means of an indirect chemiluminescence immuno-assay (CLIA) using the test kits LIAISON^®^ VZV IgG, LIAISON^®^ Measles IgG, and LIAISON^®^ Mumps IgG (all distributed by DiaSorin S.p.A., Saluggia, Italy). Measurements were performed following the manufacturer’s recommendations. Briefly, during step 1 of the incubation, specific virus IgG antibodies are bound to solid phase magnetic particles coated with the specific viral antigen. A monoclonal mouse anti-human IgG conjugated with isoluminol binds to the IgG/viral antigen-conjugate during step 2 of the incubation. Unbound materials are removed by washing after each incubation. After chemiluminescence is started by adding the reagents included in the kit, the light emitted is proportional to the specific viral IgG concentration and can be detected by a photomultiplier (LIAISON^®^ Analyzer).

Results of blood serum VZV IgG concentration were expressed as micro-international units per ml (mIU/ml). Following the recommendations of the STIKO (27), VZV-IgG concentrations below 50 mIU/ml were classified as negative, and concentrations ranging 50–100 mIU/ml were classified as borderline but interpreted as negative, while concentrations above 100 mIU/ml were classified as positive.

Results of blood serum measles and mumps IgG concentrations using the LIAISON^®^ Analyzer were expressed as arbitrary units per ml (AU/ml). Measles-IgG concentrations below 13.5 AU/ml were classified as negative, while concentrations above 16.5 AU/ml were classified as positive. Concentrations ranging 13.6–16.5 AU/ml were classified as borderline and were retested to confirm the result. Mumps-IgG concentrations below 9.0 AU/ml were classified as negative, while concentrations above 11.0 AU/ml were classified as positive. Concentrations ranging 9.0–11.0 AU/ml were classified as borderline and were retested to confirm the result.

Concerning borderline results in first-time measles or mumps measurements, specimens measured for the second time in the positive range were finally classified as positive, and specimens in the negative range were finally classified as negative. When retested, specimens in the borderline range were classified as borderline results.

Titers against rubella were determined from a blood serum specimen by means of a two-step chemiluminescence microparticle immuno-assay (CMIA) using the test kit ARCHITECT^®^ Rubella IgG (Abbot Diagnostics, Sligo, Ireland). Measurements were performed following the manufacturer’s recommendations. Briefly, during step 1 of the incubation, rubella virus IgG antibodies are bound to solid phase paramagnetic microparticles coated with rubella viral antigens. A monoclonal mouse anti-human IgG conjugated with acridine binds to the IgG/rubella antigen-conjugate during step 2 of the incubation. Unbound materials are removed by washing after each incubation. After chemiluminescence is started by adding the trigger reagents included in the kit, the light emitted is proportional to the specific viral IgG concentration and can be detected by a photomultiplier (ARCHITECT iSystems). Results were expressed as IU/ml. Rubella-IgG concentrations below 4.9 IU/ml were classified as negative, while concentrations above 10.0 IU/ml were classified as positive. Concentrations ranging 5.0–9.9 IU/ml were classified as borderline.

### Treatment of CML and Stage of Disease in the Individual Patients Prior to Vaccination

Patient #1 is a 12-year-old girl, diagnosed with CML-CP in July 2015. Following treatment with imatinib at the recommended dose of 400 mg daily, she achieved the milestones of response according to the European LeukemiaNet (ELN) recommendations at 1 year of treatment (28). She was vaccinated in July 2017 (see Results section). After 3 years (July 2018) of treatment, she lost major molecular remission. Therefore, she was switched to dasatinib (60 mg/sqm^2^ daily) in August 2018. Six months after the switch, a deep molecular response (ratio BCR-ABL1/ABL1 = 0.01%) was reached.

Patient #2 is a girl who presented at the age of 11 years with a diagnosis of CML-CP in August 2011. She was treated with imatinib at the recommended dose and achieved the milestones of response following the ELN recommendations (28). She was vaccinated in July 2012 (see section “Results”). In August 2017, CML treatment failure (cytogenetic relapse) was diagnosed. This prompted a switch to dasatinib, which was not effective, as bone marrow cytogenetic analysis showed increasingly Ph+ chromosomal interphase nuclei (11%) in November 2017. Following another switch of treatment to nilotinib, the patient underwent alloSCT from a matched unrelated donor in May 2018. At the last follow-up, 16 months after transplant (September 2019), she is alive and well and in major molecular remission without graft versus host disease.

Patient #3 was diagnosed with CML-CP in January 2016 when he was 14 years old. He was treated with the recommended dose of 400 mg imatinib daily and required several interruptions and dose modifications due to hematologic toxicity. He was vaccinated in February 2017 (see section “Results”). He did not achieve the milestones of response as recommended by the ELN (28) and therefore was switched to dasatinib treatment (70 mg daily) after 15 months in March 2017. When MR was lost in July 2017, treatment of CML was changed by switching to nilotinib. In September 2017, the boy underwent alloSCT from his HLA-identical sibling. He is alive but still affected by chronic GVHD at the last follow-up (November 2019) examination.

Patient #4 was diagnosed with CML-CP at the age of 5 years in July 2009. He was treated with the age-adapted recommended dose of 200 mg imatinib and achieved major molecular response according to the milestones recommended by the ELN (28). He was vaccinated in May 2016 (see section “Results”) when his minimal residual disease level was at 0.02% BCR-ABL1/ABL1, according to the international scale. Different from the other three cases, it was decided to interrupt the TKI treatment for a total of 3 weeks prior and post-vaccination. In July 2018, the TKI dose was adjusted to 400 mg, reflecting an increased body surface. The boy is alive and well and has been in ongoing major molecular remission as of June 2019.

## Results

### Details on Vaccination in Individual Cases

The vaccination status of patient #1 was largely incomplete at diagnosis. Except for one single vaccination at the age of 18 months with Hexavac^®^, no further vaccinations had been performed. Any infections with either measles, mumps, rubella, and varicella were not remembered, and in line with this anamnesis at diagnosis of CML, her varicella and rubella serum titers were negative. However, measles IgG (84 IU/ml) was positive and mumps IgG (11 U/ml) was in the borderline range. When rechecked prior to vaccination in March 2017, varicella and rubella titers remained negative.

In July 2017, while on imatinib treatment, vaccination was performed with M-M-RVAX Pro^®^ and simultaneously with Varivax^®^. Total lymphocyte blood count was 3,400/μl. These vaccinations were well tolerated and followed by an uneventful course. In February 2018, a second (boost) vaccination with Priorix-Tetra^®^ was administered at a blood total lymphocyte count of 3,600/μl without acute or late adverse events. Serum titer conversion was confirmed in July 2018 with positive varicella IgG (638 mIU/ml) and rubella IgG (89 IU/ml). At the last visit in May 2019, she had maintained high protective measles, mumps, and varicella titers on dasatinib therapy, while the CML was well controlled at the level of deep molecular response.

In patient #2, vaccination status was incomplete, as she had received only a single shot of measles/mumps/rubella vaccination at the age of 15 months. There was no history of varicella infection. In line with this, at diagnosis of CML, her varicella IgG titer was negative as well as her serum titers against measles and mumps, while rubella was positive (19 IU/ml).

In July 2012, while on imatinib treatment, vaccination was performed with Priorix^®^. Her total lymphocyte count was 2,330/μl. Vaccination was well tolerated and followed by an uneventful course. However, serum titer conversion could not be confirmed, as measles titer was determined negative in March 2013 as well in November 2014, while rubella and mumps titers were not determined. She was revaccinated with Priorix^®^ in July 2015 at a total lymphocyte count of 1,800/μl, which resulted in a positive serum IgG titer (114 IU/ml) against measles in October 2015. Vaccination against varicella with Varilix^®^ was performed at a total lymphocyte count of 1,760/μl in February 2016 but failed to induce seroconversion when assessed in June 2017 and February 2018. At the latter point in time, the measles titer also weaned into the borderline range (12 IU/ml).

Patient #3 had been vaccinated according to the German national immunization schedule developed by the Standing Committee on Vaccination (STIKO). At diagnosis of CML, the titers against varicella and measles were protective (197 mIU/ml and 118 IU/ml, respectively), while his titer against mumps was in the borderline range (10 IU/ml) and the titer against rubella was negative. In February 2017, while still on imatinib treatment, he was vaccinated with Priorix^®^. The total lymphocyte count was 1,475/μl. The vaccination was well tolerated and followed by an uneventful course. When reassessed in July 2017, his serum rubella titer had converted to protective titer (19 IU/ml).

Patient #4 had never been vaccinated, and at the age of 12 years (84 months after diagnosis in May 2016), titers against measles, mumps, and rubella were negative. Different from the approach in the other three patients, imatinib was stopped prior to vaccination, as the minimal residual disease levels of leukemic cells were very low. One week thereafter, he was vaccinated with Priorix^®^ while the lymphocyte count was 1,725/μl. Imatinib treatment was resumed 2 weeks after the vaccination, which was well tolerated without observed side effects. Protective seroconversion was confirmed 1 month later.

However, 10 months later (March 2017), protective titers against measles were lost, while titers against mumps and rubella were not determined. Revaccination with Priorix^®^ was performed in March 2017. Similar to the approach of the first vaccination, imatinib treatment was interrupted 1 week before and was resumed 2 weeks after the vaccination. This re-vaccination was also tolerated without side effects and resulted in protective titers against measles, mumps, and rubella. Annual reassessment of vaccination titers has been performed, and titers so far have remained protective.

All seven live virus vaccinations in the four patients (see Table 1) were well tolerated, and no acute or late adverse events were observed. All patients exhibited IgG serum levels in the normal range, and also, lymphocyte counts were above 1,500 cells/μl when assessed prior to vaccination. No comedication besides imatinib was administered to all four patients in the week prior to vaccination and 4 weeks thereafter.

**TABLE 1:** Details on the four patients receiving live virus vaccines at seven time points while on imatinib treatment.

Patient number/Gender/Age at diagnosis of CML	Age at vaccination	Time after diagnosis of CML/disease status at vaccination	Serology status prior to vaccination^1^	Lymphocyte count at vaccination	Vaccination against^2^	Serology status post vaccination	Outcome of vaccination
#1/Female/12 years	14 years	24 months CCyR No MR3.0	Measles IgG pos, Mumps IgG borderline, Rubella IgG neg, Varicella IgG neg	3400/μl	Measles, Mumps, Rubella, Varicella	Measles IgG pos, Mumps IgG pos, Rubella IgG pos, Varicella IgG pos	No acute or late adverse events, stable seroconversion achieved when assessed 12 mos after vaccination
#2/Female/11 years	12 years	11 months CCyR MR3.0	Measles IgG neg, Mumps IgG neg, Rubella IgG pos, Varicella IgG neg	2330/μl	Measles, Mumps, Rubella	Measles IgG neg, Mumps not done, Rubella not done	No acute or late adverse events, seroconversion against measles not achieved when assessed 6 mos after vaccination
	15 years	47 months CCyR MR3.0	Measles IgG neg, Mumps not done, Rubella not done	1800/μl	Measles, Mumps, Rubella	Measles IgG pos, Mumps not done, Rubella not done	No acute or late adverse events, seroconversion against measles achieved when assessed 3 mos after vaccination but lost again when assessed at 30 mos
	15 years	54 months CCyR MR3.0	Varicella IgG neg	1760/μl	Varicella	Varicella IgG neg	No acute or late adverse events, seroconversion against varicella not achieved when assessed 5 mos after vaccination
#3/Male/14 years	15 years	18 months CCyR No MR3.0	Measles IgG pos, Mumps IgG borderline, Rubella IgG neg, Varicella IgG pos	1745/μl	Measles, Mumps, Rubella	Measles IgG pos, Mumps IgG pos, Rubella IgG pos, Varicella IgG pos	No acute or late adverse events, seroconversion achieved when assessed 5 mo after vaccination
#4/Male/5 years	12 years	84 months CCyR MR3.0	Measles IgG neg, Mumps IgG neg, Rubella IgG neg	1725/μl	Measles^3^, Mumps^3^, Rubella^3^	Measles IgG pos, Mumps IgG pos, Rubella IgG pos	No acute or late adverse events, seroconversion achieved when assessed 1 mos after vaccination but lost again 10 mos after vaccination
	13 years	94 months CCyR MR3.0	Measles IgG neg, Mumps not done, Rubella not done	Not done	Measles^3^, Mumps^3^, Rubella^3^	Measles IgG pos, Mumps IgG pos, Rubella IgG pos	No acute or late adverse events, seroconversion achieved when assessed 12 mos after vaccination

*^1^For details on positive (protective), negative, and borderline serum IgG titers see section “Patients and Methods.” ^2^Commercially available vaccines used are listed in the section “Patients and Methods.” ^3^Different from patients #1, #2, and #3 in patient #4 the TKI treatment with imatinib was interrupted 1 week prior and 2 weeks after vaccination. For details see text. CML, chronic myeloid leukemia; CCyR, complete cytogenetic remission of CML (no detectable Philadelphia chromosome); MR3.0, molecular remission at a ratio of 0.1% BCR-ABL1/ABL1 transcripts on the International Scale; mos, months.*

## Discussion

Measles is a highly contagious viral disease with an infectivity rate close to 100%. In the United States and in European countries, the number of reported cases of measles is increasing, reflecting a global rise that began in 2018 and still continues (29–33). As of today, WHO’s goal of global elimination of measles has not been achieved. Previous gains are now counteracted by a 31% increase in the number of measles cases reported globally between 2016 and 2017 (34). In Europe, the number of reported cases of measles in 2018 was triple of those reported in 2017 and 15 times of those in 2016 (31). It must be feared that endemic measles has now been re-established in several European countries where transmission has previously been interrupted (35). Worldwide, the 10 countries with the highest numbers of cases reported in 2019 include Brazil, India, Kazakhstan, Madagascar, Nigeria, Pakistan, Philippines, Thailand, Ukraine, and Venezuela (31, 32). The severity of the problem is highlighted by WHO’s report of 140,000 measles deaths in 2018 (35). Patients with CML undoubtedly are in need of protective serum titers, not only when traveling to these countries, either because of vacation plans or because of occupational obligations, but also when facing a local outbreak close to their living area.

According to the general recommendations that are also provided by self-help organizations via the internet (36), individuals with CML should not receive the measles/mumps/rubella live vaccine unless approved by their CML specialist. To improve herd immunity, it is safe, however, for family members or for those in close contact with CML patients to receive vaccination. In addition, it has been established that patients with CML who are exposed to someone who has varicella or shingles disease or measles should alert their healthcare team, which will consider if they are quickly eligible to receive chemoprophylaxis with acyclovir or hyperimmune globulin. Of note, immunosuppressed cancer patients may present with atypical severe complications that have not been documented in immunocompetent patients, such as giant-cell pneumonia, measles inclusion-body encephalitis, and abdominal pain due to visceral dissemination and fulminant hepatitis in the case of varicella infection (37, 38). In outbreaks of varicella, measles, or influenza on oncological ward or stem cell transplant units or in renal transplantation, a 5–10% case fatality rate has been reported (39–45).

The established live vaccines (measles, mumps, rubella, and yellow fever) have long been generally contraindicated for immunocompromised patients, as the live attenuated virus may cause disease depending on the residual capacity of the host’s immune system. Pharmaceutical manufacturers of live vaccines contraindicate vaccination of immunosuppressed individuals in general, without any differentiation (46, 47). Thus, immunosuppressed people must rely on herd immunity to protect themselves from infection. However, the intensity varies with which a given immunosuppressive therapy (e.g., acute leukemia induction treatment versus juvenile rheumatic patients receiving methotrexate or corticosteroids chronically) will attenuate the immune response. In particular, the dosages and the administration schedule of immunosuppressants have an influence on the grade of immunosuppression. Thus, assessment of the degree of immunodeficiency requires expert knowledge. In more recent expert statements, the recommendations for vaccination were developed on the basis of current evidence and theoretical/immunological considerations, which classified immunosuppression into three stages, i.e., no relevant immunosuppression, mild to moderate, and severe immunosuppression (48–50). Assigning different immunosuppressant drugs (including biologicals) to one of the three stages allows for a more tailored approach when considering the safety and efficacy of attenuated live vaccines in immunocompromised patients.

As an example, varicella vaccine was found to be safe in patients with inflammatory bowel disease on immunosuppressive therapies (51, 52), in juvenile rheumatic patients receiving methotrexate or corticosteroids, and in pediatric patients with acute lymphoblastic leukemia on maintenance treatment (weekly methotrexate plus daily 6-mercaptopurine) (53, 54). The results we have so far suggest that live vaccines may be safe in some patients receiving long-term immunosuppressive therapies. However, this topic remains controversial, with still very limited data available to support a general recommendation (48).

In patients with CML, during the first 6–12 months of TKI treatment, the white blood cell counts, including the lymphocyte counts, may vary considerably due to the off-target inhibition of the stem cell factor c-kit. Guidelines provide steering criteria of when to interrupt and when to restart CML therapy with TKI, based on the peripheral blood cell counts (28). The threshold of 1,500 lymphocytes/μl is generally considered the lowest count for a sufficient immune response to a live vaccine (6). Therefore, it might be prudent to postpone any live vaccines until after the first year of TKI treatment when blood counts show less fluctuation.

Evidently, the four patients presented here represent an inhomogeneous cohort with regard to immunonaivety. Except for patient #4, who had never been vaccinated and exhibited no viral IgG titers prior to vaccination (see Table 1), the other three patients were partly seropositive, either due to an infection they did not recollect (in patient #1, measles IgG borderline) or an incomplete vaccination schedule (in patient #2, after a single shot of Priorix^®^ at the age of 15 months: measles and mumps IgG negative, but rubella IgG positive), or had a complete vaccination schedule (in patient #3: measles and varicella IgG positive, mumps IgG borderline, and rubella IgG negative). The finding of missing seropositivity or borderline IgG values as determined in patient #1 after 24 months, in patient #2 after 11 months, and in patient #3 after 18 months of imatinib treatment, respectively, may be in line with reports pointing to impaired memory B-cell numbers, as well B-cell immune responses under imatinib exposure, when adults with CML were compared to age-matched healthy controls (21). In the four patients described in this report, besides determination of IgG titers, no more detailed analyses were performed.

Furthermore, the investigation is weakened by a non-systematic assessment of seroconversion. While seroconversion could be confirmed in patients #1, #3, and #4, in patient #2, the first vaccination against measles, mumps, and rubella performed 11 months after diagnosis of CML was shown to be unsuccessful when assessed 6 months after vaccination. Revaccination 3 years later resulted in an initial seroconversion after 3 months but was lost again in the course of CML treatment. Of note, the latter course was similarly observed in patient #4 who exhibited seroconversion when assessed 1 month after vaccination but lost protective titer against measles when assessed 10 months thereafter. Therefore, in patients assessed late after vaccination, it is not known if they failed to respond or if they responded but the response was not maintained.

When vaccination in patient #2 against varicella resulted in no measurable VZV-IgG in serum in the 54th month of imatinib treatment, it must be remembered that a missing detection of VZV IgG is observed in 1–5% of healthy vaccinees (55) and may be caused by acquired T-cellular immunity against varicella (not investigated in this case). In addition, standard rubella IgG measurements can be false-negative due to the high-neutralizing antibodies of the vaccinee.

Patient #4 differs from the other three patients because, for both vaccinations, the imatinib treatment was interrupted from 1 week prior to the vaccination to 2 weeks thereafter. Short treatment interruptions are acceptable if the minimal residual disease burden is at a low level (ratio BCR-ABL1/ABL1 = 0.1% or lower). While a time period of 1 week suffices to wash out imatinib based on the pharmacokinetic data on the drug’s serum half-time (56, 57), the interval of 2 weeks post-vaccination for the development of an immune response might have been rather short. When more than 200 children aged 9 months were vaccinated for measles, IgM and IgG positivity rates of 61 and 14%, respectively, at 2 weeks post-vaccination were observed (58). Therefore, being off TKI for an additional time post-vaccination might be more efficient, but one must keep in mind that relapse of CML can occur very rapidly after cessation of TKI treatment (59). This is why the monitoring of minimal residual disease at 4-week intervals is recommended for CML in most TKI-stopping studies, thus underscoring that MR might be lost within a couple of weeks (9, 10, 15). For that reason, when we did not interrupt the TKI treatment of CML to perform vaccination for a prolonged interval, we accepted a lower seroconversion rate in favor of minimizing the relapse risk.

We are well aware that the number of patients presented here is much too low to draw definite conclusions, and the observations described may hold true for the TKI imatinib only. Dasatinib has a different immunosuppressive profile, and little to nothing is known on the other second- or third-generation TKIs—neither in children, nor in adults. Future research is undoubtedly necessary, and a number of open questions remain: What is the effect of different TKI medications on virus-specific cell-mediated and humoral immunity over time? Who should have the potentially immunosuppressive TKI treatment delayed, to allow for vaccination? Is delaying low-risk immunosuppression necessary in the context of receiving a live vaccine, and does it affect the likelihood of seroconversion?

## Conclusion

As long as herd immunity against dangerous diseases like measles is not established in many countries, patients with CML require protection. No definitive conclusions can be drawn from the small number of cases presented here, other than that live vaccination was well-tolerated without acute or long-term side effects in all four patients. However, from a theoretical viewpoint, live virus vaccination in patients on imatinib may be considered on an individual basis if the following prerequisites are fulfilled:

i) The patient is living in or traveling to an area with poor herd immunity.
ii) The patient is in a stable phase of CML (cytogenetic response, ratio BCR-ABL1/ABL1 = 1% or lower expressed on the international scale, stable lymphocyte counts >1,500 μl. In the near-future, hematologic changes caused by TKI treatment or switching to another TKI are not to be expected).
iii) Prior vaccination with an inactivated vaccine has resulted in an adequate immune response.
iv) The benefits and risks of vaccination are discussed in depth with the patient and his/her legal guardians, and written informed consent is obtained.

In order to accumulate knowledge, we also suggest that any experience with live virus vaccines in children with CML should be documented in an international CML registry.

## Data Availability Statement

All datasets generated for this study are included in the article/supplementary material.

## Ethics Statement

The studies involving human participants were reviewed and approved by the Ethical Board of the Medical Faculty of the Technical University of Dresden (Ethical vote # EK282122006). Written informed consent to participate in this study was provided by the participants’ legal guardian/next of kin.

## Author Contributions

The concept of this investigation was developed by all of the authors. CBC-R and MN treated the patients, communicated the risks and benefits of each live virus vaccination with the patients and their parents, and sought informed consent for this “off-label” approach. VH performed all data collection and data management. MM and MS supervised all therapeutic approaches, wrote the first draft of the manuscript, and critically revised all comments. All of the authors revised the text and approved the final version of the manuscript.

## Conflict of Interest

The authors declare that the research was conducted in the absence of any commercial or financial relationships that could be construed as a potential conflict of interest.
